# Glucagon-like peptide-1 (GLP-1) mediates cardioprotection by remote ischaemic conditioning

**DOI:** 10.1093/cvr/cvw216

**Published:** 2016-10-04

**Authors:** Marina V. Basalay, Svetlana Mastitskaya, Aleksander Mrochek, Gareth L. Ackland, Ana Gutierrez del Arroyo, Jenifer Sanchez, Per-Ove Sjoquist, John Pernow, Alexander V. Gourine, Andrey Gourine

**Affiliations:** 1 Centre for Cardiovascular and Metabolic Neuroscience, Neuroscience, Physiology & Pharmacology, University College London, Gower Street, London WC1E 6BT, UK;; 2 Research Centre Cardiology, Luxemburg Street 110, Minsk 220026, Belarus;; 3 William Harvey Research Institute, Queen Mary University of London, Charterhouse Square, London EC1M 6BQ, UK; and; 4 Karolinska Institute, Division of Cardiology, Karolinska University Hospital, Solna 171 76, Stockholm, Sweden

**Keywords:** Cardioprotection, Glucagon-like peptide-1, Myocardial infarction, Myocardial ischaemia, Parasympathetic, Remote ischaemic conditioning, Reperfusion, Vagus nerve

## Abstract

**Aims:**

Although the nature of the humoral factor which mediates cardioprotection established by remote ischaemic conditioning (RIc) remains unknown, parasympathetic (vagal) mechanisms appear to play a critical role. As the production and release of many gut hormones is modulated by the vagus nerve, here we tested the hypothesis that RIc cardioprotection is mediated by the actions of glucagon-like peptide-1 (GLP-1).

**Methods and results:**

A rat model of myocardial infarction (coronary artery occlusion followed by reperfusion) was used. Remote ischaemic pre- (RIPre) or perconditioning (RIPer) was induced by 15 min occlusion of femoral arteries applied prior to or during the myocardial ischaemia. The degree of RIPre and RIPer cardioprotection was determined in conditions of cervical or subdiaphragmatic vagotomy, or following blockade of GLP-1 receptors (GLP-1R) using specific antagonist Exendin(9–39). Phosphorylation of PI3K/AKT and STAT3 was assessed. RIPre and RIPer reduced infarct size by ∼50%. In conditions of bilateral cervical or subdiaphragmatic vagotomy RIPer failed to establish cardioprotection. GLP-1R blockade abolished cardioprotection induced by either RIPre or RIPer. Exendin(9–39) also prevented RIPre-induced AKT phosphorylation. Cardioprotection induced by GLP-1R agonist Exendin-4 was preserved following cervical vagotomy, but was abolished in conditions of M3 muscarinic receptor blockade.

**Conclusions:**

These data strongly suggest that GLP-1 functions as a humoral factor of remote ischaemic conditioning cardioprotection. This phenomenon requires intact vagal innervation of the visceral organs and recruitment of GLP-1R-mediated signalling. Cardioprotection induced by GLP-1R activation is mediated by a mechanism involving M3 muscarinic receptors.

## 1. Introduction 

Powerful innate mechanisms of cardioprotection can be recruited by remote ischaemic conditioning (RIc), which can be established by cycles of ischaemia/reperfusion applied to an organ/tissue distant from the heart. In several animal models significant reduction of myocardial ischaemia/reperfusion injury was demonstrated when RIc stimulus was applied either before (remote ischaemic preconditioning, RIPre) or during myocardial ischaemia (remote ischaemic perconditioning, RIPer),^1^^,^^2^ or after the onset of reperfusion (remote ischaemic postconditioning).^3^ Clinical trials have demonstrated the efficacy of RIc in reducing infarct size in patients with an acute myocardial infarction (AMI),^4^^,^^5^ in reducing myocardial damage during cardiac surgery^6^ and improving long term prognosis in both patient cohorts.^6^^,^^7^ Although the exact mechanisms underlying RIc cardioprotection are not fully understood, a number of studies suggested the involvement of both humoral^8–11^ and neural signalling pathways.^1^^,^^12–14^ Several candidate humoral factors of RIc have been proposed, including stromal cell-derived factor-1α,^15^ nitrite/nitric oxide,^16^ interleukin-10,^17^ microRNA-144,^18^ apolipoprotein A-I^19^ and alpha-ketoglutarate-dependent dioxygenase Egln1.^20^ However, the full extent of RIc cardioprotection cannot be explained by the actions of any of these factors alone.

There is strong evidence that parasympathetic (vagal) mechanisms are critically important for RIPre cardioprotection. RIPre was reported to be abolished by selective genetic inhibition of brainstem vagal preganglionic neurones,^13^ muscarinic receptor blockade,^13^^,^^14^ bilateral cervical vagotomy^3^ or sectioning of the posterior gastric branch of the vagus nerve.^21^ Electrical stimulation of the whole vagus nerve at the cervical level^22^^,^^23^ or isolated posterior gastric branch of the vagus^21^ establishes cardioprotection. These data suggest that visceral organs, innervated by the posterior gastric branch of the vagus nerve, are the likely source of a humoral factor (or factors) of RIc cardioprotection. Glucagon-like peptide-1 (GLP-1) is the most notable of all humoral factors, which originate from the visceral organs and known to have cardioprotective properties. GLP-1 is an incretin hormone released by the L-cells of the intestine in response to the ingestion of food.^24^^,^^25^ Release of GLP-1 is modulated by vagal efferent (motor) activity^26^^,^^27^ and there is also evidence that GLP-1 may interact with vagal sensory fibres innervating the viscera.^26^ GLP-1 actions appear to be mediated via glucagon-like peptide-1 receptor (GLP-1R)-dependent and independent mechanisms,^28^^,^^29^ although the existence of the latter is debated. Studies conducted in animal models demonstrated potent cardioprotection by GLP-1R activation.^30^ The efficacy of GLP-1R agonists in reducing infarct size was also shown in human studies.^31^^,^^32^ Recent study conducted in patients with type 2 diabetes demonstrated significant reduction in frequency of adverse cardiovascular events and death from cardiovascular causes by treatment with GLP-1 analogue liraglutide.^33^ The molecular weight of GLP-1 is ∼3.3 kDa and it appears to satisfy the key criteria of the humoral preconditioning factor (including molecular weight of less than 8 kDa), suggested by Lang and colleagues on the basis of the proteomic analysis of blood samples obtained from experimental animals receiving the RIPre stimulus.^34^

This study was designed to test the hypothesis that cardioprotection established by RIc is mediated by the actions of GLP-1. Taken into the account that the parasympathetic nervous system appears to be critically important for RIPre cardioprotection, we also investigated the significance of vagal mechanisms in establishing cardioprotection induced by the GLP-1R agonist Exendin-4 (Ex4).

## 2. Methods

All the experiments were performed in accordance with the European Commission Directive 2010/63/EU (European Convention for the Protection of Vertebrate Animals used for Experimental and Other Scientific Purposes) and the UK Home Office (Scientific Procedures) Act (1986) with project approval from the respective Institutional Animal Care and Use Committees.

### 2.1 Animal preparation

Adult male Sprague Dawley rats (280–320 g) were anaesthetized with pentobarbital sodium (induction 60 mg kg^−1^ i.p.; maintenance 15 mg kg^−1^ h^−1^ i.v.). Adequate anaesthesia was ensured by maintaining stable levels of the arterial blood pressure and heart rate and confirmed by the absence of a withdrawal response to a paw pinch. The right carotid artery and left jugular vein were cannulated for the measurement of the arterial blood pressure and administration of anaesthetic or test compounds, respectively. The trachea was cannulated to allow mechanical ventilation with room air using a positive pressure rodent ventilator (tidal volume ∼8–10 ml kg^−1^; frequency ∼60 strokes min^−1^). Partial pressures of O_2_ and CO_2_ as well as pH of the arterial blood were measured regularly. A standard lead II ECG was recorded. The body temperature was maintained at 37.0 ± 0.5 °C with a servo-controlled heating blanket.

### 2.2 Model of myocardial infarction

An established rat model of myocardial ischaemia/reperfusion injury was used.^3^^,^^13^^,^^21^ The heart was exposed via a left thoracotomy and a 5–0 monofilament polypropylene suture was passed around the left anterior descending coronary (LAD) artery to induce a temporary occlusion. LAD artery was occluded for 30 min followed by reperfusion lasting 120 min.

### 2.3 Infarct size measurement

At the end of the reperfusion period, the LAD artery was ligated, and 5% Evans Blue dye solution (0.2 ml) was infused via the jugular vein to determine the area at risk. The animal was then given an anaesthetic overdose (pentobarbital, 200 mg kg^−1^, i.v.), the heart was excised, the left ventricle (LV) was isolated, frozen, and sectioned into 5–6 transverse slices from the apex to the base. The area at risk was demarcated by the absence of Evans Blue staining. LV slices were then incubated with 1% 2,3,5-triphenyltetrazolium chloride (TTC) in Tris buffer (pH 7.4) for 15 min at 37 °C and fixed in 4% formalin for 24 h. Viable myocardium is stained red by TTC, whereas necrotic myocardium appears white. The area at risk and the necrotic area were determined by computerized planimetry, normalized to the weight of each slice, with the degree of necrosis (i.e. infarct size) expressed as the percentage of area at risk, as described.^3^^,^^13^^,^^21^

### 2.4 Immunoblotting

For the analysis of protein phosphorylation, Western blot was performed on the myocardium from the area at risk. The ventricular tissue was excised, frozen in liquid nitrogen and stored at −80 °C before the assays. Total phosphatidylinositol 3-kinase AKT (PI3K/AKT), phospho-AKT (Ser473), janus-activated kinase (JAK) signal transducer and activator of transcription (STAT3) and phospho-STAT3 (Tyr705) were immunodetected in cell lysates (whole cell fractions) using specific primary antibodies (all from Cell Signalling Technology, UK). Proteins were electrophoretically separated in SDS-PAGE gels and transferred to polyvinylidene difluoride membranes (Amersham Biosciences, USA) according to the manufacturer’s instructions. After antibody labelling, detection was performed (ECL detection system, Amersham Biosciences, USA). Densitometry was used to calculate the ratio of phosphorylated and total protein normalized to the expression of β-actin (Santa Cruz UK) to control protein loading.

### 2.5 Experimental protocols

#### 2.5.1 Experiment 1. The effect of vagotomy on RIPer cardioprotection

**Figure 1:**
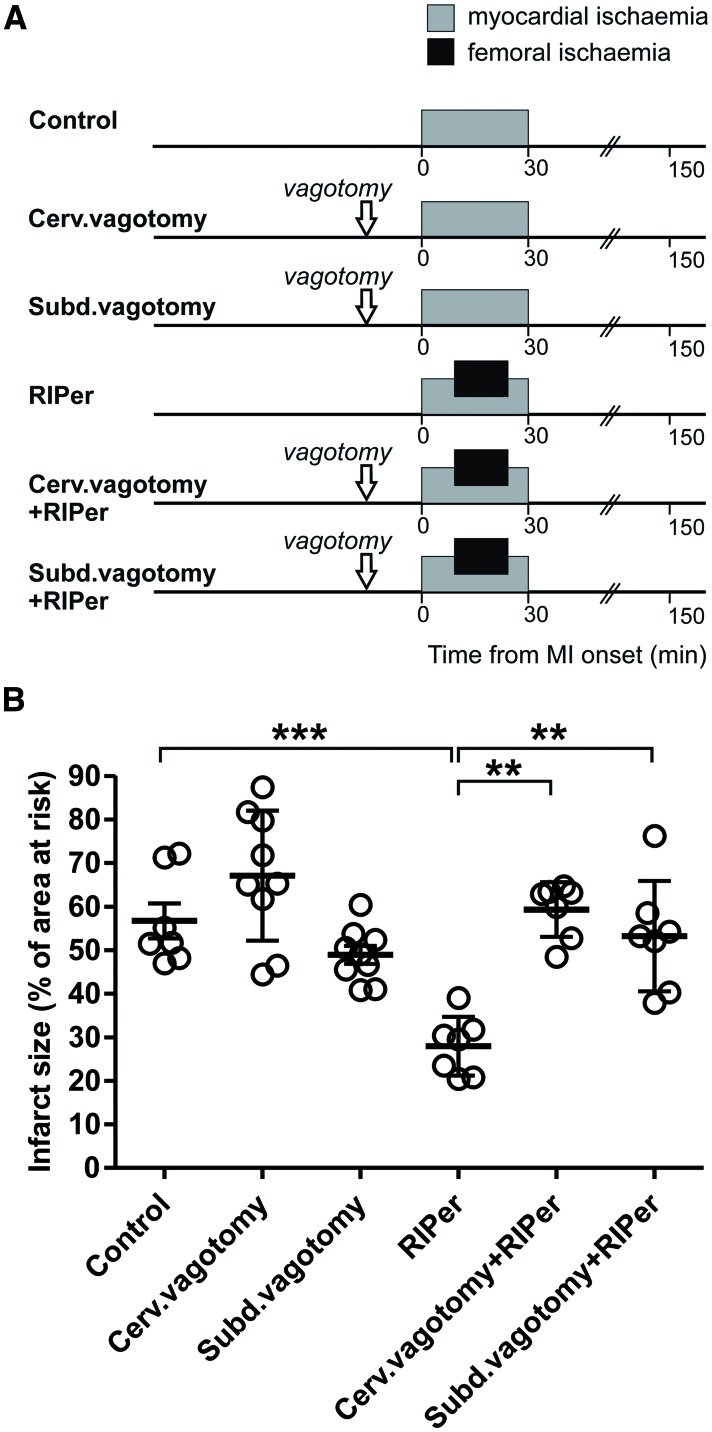
Cardioprotection induced by remote ischaemic perconditioning requires intact parasympathetic innervation of visceral organs. (*A*) Illustration of the experimental protocols. In all the protocols, the rat model of myocardial infarction involved 30 min of left anterior descending coronary artery occlusion followed by 120 min of reperfusion. Arrows indicate time (15 min before myocardial ischaemia) of bilateral cervical (cerv.) or subdiaphragmatic (subd.) vagotomy. Remote ischaemic perconditioning (RIPer) was induced by occlusion of both femoral arteries for 15 min starting 10 min after the onset of myocardial ischaemia. (*B*) Infarct size is presented as a percentage of the area at risk. Individual data and means ± SD are shown. **P < 0.01; ***P < 0.001.

There is evidence that RIPre cardioprotection is abolished in conditions of either cervical^3^ or subdiaphragmatic^21^ vagotomy. However, the role of parasympathetic mechanisms in mediating RIPer cardioprotection remains unknown. RIPer was induced by occlusion of both femoral arteries for 15 min starting 10 min after the onset of myocardial ischaemia (see timeline *Figure 1A*). For bilateral cervical vagotomy, both nerves were exposed at the neck level and sectioned 15 min prior to myocardial ischaemia. To perform total subdiaphragmatic vagotomy, minimal incision was made to gain access to the abdominal cavity, the left lobes of the liver were gently pulled aside, the stomach was retracted caudally to expose the oesophagus, the vagal trunks were carefully dissected under the diaphragm and sectioned.^21^^,^^35^

#### 2.5.2 Experiment 2. The effect of GLP-1 receptor blockade on RIc-induced cardioprotection and phosphorylation of AKT and STAT3

**Figure 2:**
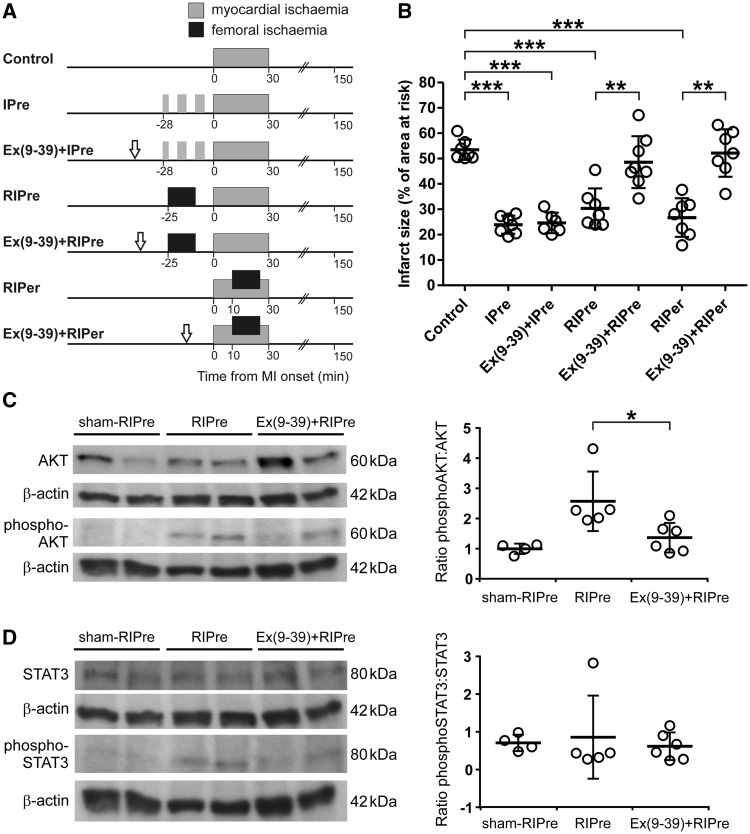
GLP-1 receptors mediate remote ischaemic conditioning cardioprotection. (*A*) Illustration of the experimental protocols. Myocardial ischaemic preconditioning (IPre) was induced by three episodes of myocardial ischaemia (3 + 5 + 5 min) separated by 5-min periods of reperfusion. Remote ischaemic preconditioning (RIPre) was induced by occlusion of both femoral arteries for 15 min starting 25 min before the onset of myocardial ischaemia. Arrows indicate the time (15 min before IPre, RIPre or myocardial ischaemia) of intravenous administration of GLP-1 receptor antagonist Exendin(9–39) (Ex(9–39)). (*B*) Infarct size is presented as a percentage of the area at risk. Individual data and means ± SD are shown. **P < 0.01; ***P < 0.001. (*C*) *Left*: representative immunoblots showing total AKT and phospho-AKT (Ser473) protein expression in left ventricular lysates at 15 min of myocardial reperfusion in rats subjected to preparative sham surgery (sham-RIPre), application of RIPre stimulus, or application of RIPre stimulus in conditions of systemic GLP-1R blockade with Ex(9–39). *Right*: summary data illustrating means ± SD of the densitometry of phospho-AKT-to-AKT ratio. *P < 0.05. (*D*) *Left*: representative immunoblots showing total STAT3 and phospho-STAT3 (Tyr705) protein expression in left ventricular lysates at 15 min of myocardial reperfusion in rats subjected to preparative sham surgery (sham-RIPre), application of RIPre stimulus, or application of RIPre stimulus in conditions of systemic GLP-1R blockade with Ex(9–39). *Right*: summary data illustrating means ± SD of the densitometry of phospho-STAT3-to-STAT3 ratio.

RIc was induced by 15 min occlusion of both femoral arteries, followed by reperfusion, starting either 25 min prior or 10 min after the onset of myocardial ischaemia (see timeline *Figure 2A*). GLP-1R antagonist Exendin(9–39) (Ex(9–39), 50 µg kg^−1^, i.v.),^36^^,^^37^ was administered 40 min or 15 min before the onset of ischaemia in animals receiving the RIPre or RIPer stimulus, respectively. The effect of Ex(9–39) on cardioprotection established by classical myocardial ischaemic preconditioning (IPre) was also determined. IPre was induced by three episodes of myocardial ischaemia (LAD occlusion; 3 + 5 + 5 min) separated by 5-min periods of reperfusion. Ex(9–39) was given 15 min before the first ischaemic episode. The dose of Ex(9–39) was selected on the basis of previously published reports.^38^ In a separate experiment the effect of Ex(9–39) on RIPre-induced phosphorylation of AKT and STAT3 in the myocardium was assessed. The hearts were collected 15 min after the onset of myocardial reperfusion.

#### 2.5.3 Experiment 3. The effect of vagotomy and systemic muscarinic receptor blockade on cardioprotection established by GLP-1 receptor activation

**Figure 3:**
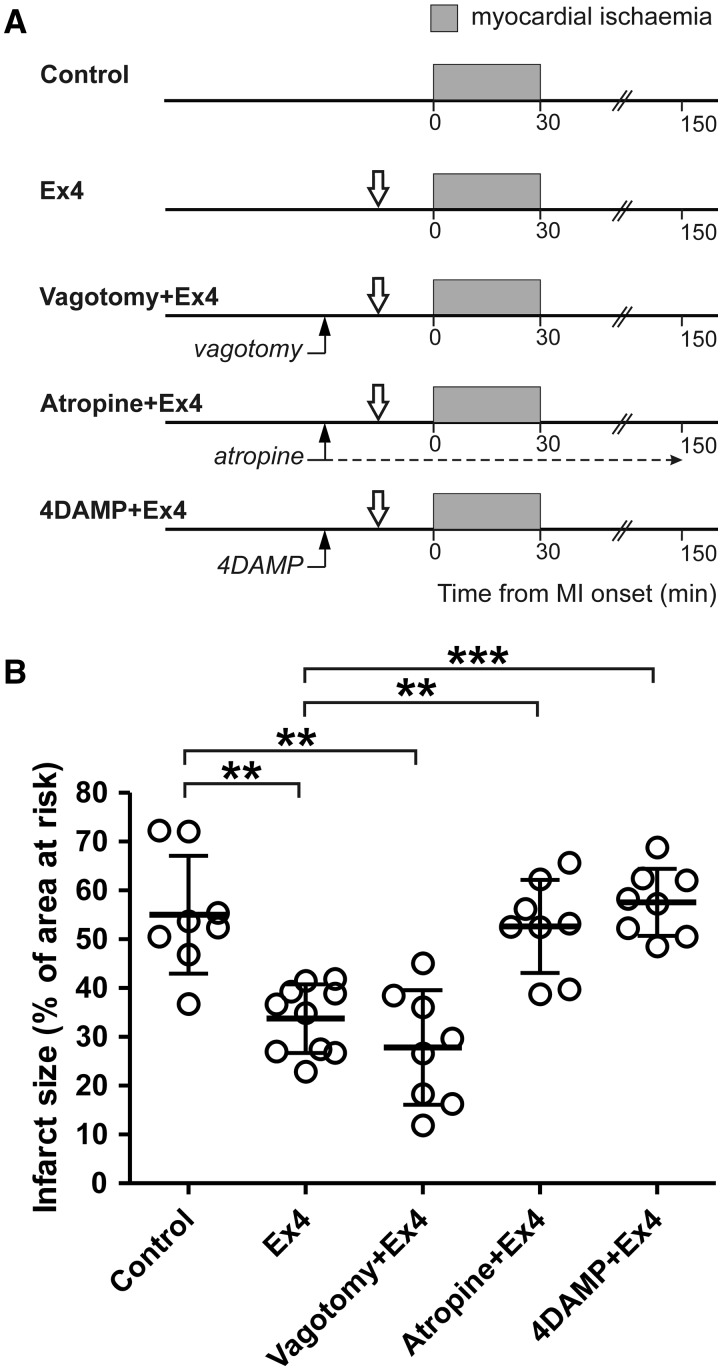
Cardioprotection induced by GLP-1 receptor activation is mediated by a muscarinic mechanism. (*A*) Illustration of the experimental protocols. Downward arrows indicate time (15 min before myocardial ischaemia) of intravenous administration of GLP-1 receptor agonist Exendin-4 (Ex4). Upward arrows indicate time (30 min before myocardial ischaemia) of cervical vagotomy, start of atropine infusion or administration of M3 muscarinic receptor antagonist 4DAMP. (*B*) Infarct size is presented as a percentage of the area at risk. Individual data and means ± SD are shown. **P < 0.01; ***P < 0.001.

Intravenous administration of GLP-1R agonist Exendin-4 (Ex4) in doses ranging between 1–10 μg kg^−^^1^ has been shown to protect the rat heart against myocardial ischaemia/reperfusion injury.^39^ In this study we used Ex4 in 5 μg kg^−^^1^ dose to determine the efficacy of GLP-1R activation in establishing cardioprotection in conditions of bilateral cervical vagotomy, systemic muscarinic receptor blockade (atropine methyl nitrate; initial bolus dose 2 mg kg^−1^, i.v., followed by infusion at a rate of 1 mg kg^−1^ h^−1^) or M3 muscarinic receptor blockade (4-DAMP; 2 mg kg^−1^, i.v). Experimental protocols are illustrated on *Figure 3A*.

#### 2.5.4 Experiment 4. The effect of RIc on plasma level of GLP-1

After 12-h overnight fast, the animals were anaesthetized and instrumented as described above. RIc was induced by 15 min occlusion of both femoral arteries, followed by reperfusion. Samples of the arterial blood (300 µl) were collected into pre-chilled EDTA-Eppendorf tubes at 4 time points: 10 min prior to and 5, 20, and 30 min after the onset of limb ischaemia or sham-RIc. Dipeptidyl peptidase-4 (DPP-4) inhibitor (50 µM; EMD Millipore) was added to the collected samples and plasma was separated by centrifugation (10 min; 1000 g; 4°C). Total GLP-1 in plasma was measured using MesoScale Discovery Total GLP-1 (v2) kit (Rockville, USA).

### 2.6 Statistical analysis

One-way ANOVA (Tukey's and Bonferroni's Multiple Comparison Tests) was used for statistical analysis of the data. Values of *P < *0.05 were considered to be significant.

## 3. Results

In the *Experiment 1*, an increase in heart rate was observed in animals subjected to RIPer in conditions of subdiaphragmatic vagotomy during myocardial ischaemia and reperfusion period (Supplementary material online, *Table*). In the *Experiment 3*, mean arterial blood pressure increased in Ex4-treated animals subjected to cervical vagotomy before myocardial ischaemia. Heart rate increased before myocardial ischaemia and remained elevated during myocardial ischaemia and during reperfusion (Supplementary material online, *Table*). No other differences in mean arterial blood pressure and heart rate during ischaemia and reperfusion were observed between the experimental groups of animals (Supplementary material online, *Table*). Areas at risk were similar between all the experimental groups (data not shown). *Figures **1**B, *2*B, and **3**B* illustrate infarct sizes expressed as percentages of the areas at risk.

### 3.1 Experiment 1. The effect of vagotomy on RIPer cardioprotection

Average infarct size in animals subjected to 30 min of LAD occlusion followed by 120 min of reperfusion was 56 ± 10% (*Figure **1**B*). RIPer significantly reduced myocardial ischaemia/reperfusion injury (infarct size 27 ± 6%, *P* < 0.001), but failed to establish cardioprotection in conditions of either bilateral cervical or subdiaphragmatic vagotomy (*Figure **1**B*). Vagotomy *per se* had no effect on myocardial ischaemia/reperfusion injury (*Figure **1**B*).

### 3.2 Experiment 2. The effect of GLP-1 receptor blockade on RIc-induced cardioprotection and phosphorylation of AKT and STAT3

To determine whether GLP-1 may act as a humoral factor of RIc we next determined the efficacy of IPre, RIPre, and RIPer in establishing cardioprotection in conditions of systemic GLP-1R blockade (*Figure **2**B*). Systemic administration of a specific GLP-1R antagonist Ex(9–39) blocked cardioprotection induced by RIPre and RIPer (infarct sizes 48 ± 10% and 52 ± 9%, respectively), but had no effect on cardioprotection conferred by classical direct myocardial IPre (infarct size 24 ± 4%) (*Figure *2*B*). RIPre-induced AKT phosphorylation was blocked (*P* < 0.05) by systemic treatment with Ex(9–39) (*Figure **2**C*). RIPre had no significant effect on STAT3 phosphorylation (*Figure **2**D*).

### 3.3 Experiment 3. The effect of vagotomy and systemic muscarinic receptor blockade on cardioprotection established by GLP-1 receptor activation

We next determined whether GLP-1R activation induces cardioprotection via recruitment of vagal mechanisms. Intravenous infusion of GLP-1R agonist Ex4 significantly reduced (by 40%, *P* < 0.01) the extent of myocardial ischaemia/reperfusion injury (*Figure **3**B*). Ex4-induced cardioprotection was not affected by bilateral cervical vagotomy (infarct size 27 ± 11%, NS vs. Ex4 treatment) (*Figure **3**B*). However, systemic muscarinic receptor blockade (atropine) abolished Ex4-induced cardioprotection (infarct size 52 ± 9%, *P* < 0.01 vs. Ex4). Ex4 also failed to establish cardioprotection in conditions of systemic M3 receptor blockade with 4-DAMP (infarct size 57 ± 6%, *P* < 0.001 vs. Ex4) (*Figure **3**B*).

### 3.4 Experiment 4. The effect of RIc on plasma level of GLP-1

Moderate increases in the level of circulating (arterial) GLP-1 compared to the baseline values were observed 30 min after the onset of limbs ischaemia (15 min into the limb reperfusion period) (4.7 ± 0.9 vs. 3.0 ± 0.7 pg/ml at baseline; *P* < 0.05; *Table **1*). Arterial GLP-1 levels were not affected by sham-RIc procedure (*Table **1*).

**Table 1:** The effect of remote ischaemic conditioning (RIc) or sham-RIc on plasma level of glucagon-like peptide-1 (in pg ml ^−^^1^)

		Time from the onset of limb ischaemia or sham (min)			
	*n*	−10	5	20	30
Sham	8	4.1 ± 0.7	5.2 ± 1.0	4.4 ± 0.9	5.1 ± 1.1
RIc	8	3.0 ± 0.7	3.1 ± 0.6	3.4 ± 0.6	4.7 ± 0.9*

* Significant difference from the baseline value (*P* < 0.05).

## 4. Discussion

To the best of our knowledge, this is the first experimental study which demonstrated that a particular humoral factor is *causally* involved in cardioprotection induced by remote ischaemic conditioning. Recent study identified the likely origin of the cardioprotective humoral factor which appears to be produced by the visceral organs innervated by the posterior gastric branch of the vagus nerve.^21^ Organs of the gastrointestinal tract indeed represent a major source of many factors with known cardioprotective properties, including GLP-1. Here we show that the remote conditioning-induced cardioprotection and phosphorylation of pro-survival kinase AKT are abolished by systemic GLP-1R blockade with Ex(9–39). Ex(9–39) has been used in many published studies, including several seminal reports, which described physiological role and significance of GLP-1R-mediated signalling,^40^ and off-target (i.e. not on GLP-1R) effects of this peptide antagonist have never been observed. The data obtained also suggest that pathways of cardioprotection downstream of GLP-1R activation are independent of vagal activity but involve recruitment of M3 receptor-dependent mechanism. These results are in agreement with the data reported recently, showing that in isolated hearts cardioprotection established by plasma dialysate collected from rats receiving the RIc stimulus is abolished by muscarinic receptor blockade.^41^

### 4.2 Vagus nerve and remote ischaemic conditioning cardioprotection

Since the importance of vagal mechanisms in mediating cardioprotection induced by RIc was first proposed,^42^ results of several experimental studies provided strong evidence in support of the idea that the intact parasympathetic mechanisms are essential for RIc cardioprotection. RIPre cardioprotection was found to be abolished by selective genetic targeting and silencing of vagal preganglionic neurones, bilateral cervical vagotomy or systemic muscarinic receptor blockade.^3^^,^^13^^,^^14^ Anatomical and functional cholinergic innervation of the cardiac ventricles was demonstrated in a number of studies (for recent experimental reports see Refs^43^^,^^44^) and it was suggested that RIPre is mediated by the actions of acetylcholine released from vagal efferent fibres which innervate the LV myocardium.^13^ However, the mechanism involving vagally-mediated reflexes cannot fully explain how RIPre is able to protect the transplanted or denervated hearts, or cross species transfer of RIc cardioprotection by plasma dialysate.^8–11^ Taken together, the available data suggest that production and release of a humoral factor of RIc cardioprotection is under parasympathetic control. In support of this idea, it was recently demonstrated that RIPre fails to establish cardioprotection in conditions of selective sectioning of the posterior gastric branch of the vagus nerve,^21^ pointing to the likely source of the cardioprotective humoral factor. Results of the present study show that RIPer cardioprotection also requires intact parasympathetic innervation of visceral organs. Therefore, the common vagal mechanisms appear to mediate cardioprotection established by RIc applied either before or during myocardial ischaemia and require GLP-1R-mediated signalling.

### 4.3 Humoral factor(s) of RIc cardioprotection. The role of GLP-1R-mediated signalling

A significant number of studies demonstrated successful transfer of humoral factor responsible for RIc cardioprotection with plasma or dialysate, obtained after RIc, to another animal or isolated hearts, even across species (see for example Refs^8^^,^^10^^,^^11^^,^^41^) The molecular weight of this humoral factor of cardioprotection appears to be less than 8 kDa,^34^ and several candidate molecules have been proposed.^15–19^ Cell-derived factor-1α,^15^ nitrite/nitric oxide,^16^ interleukin-10,^17^ microRNA-144^18^ were shown to be involved in cardioprotection; however, RIc cardioprotection cannot be fully explained by any of the identified factors acting alone. In addition, SDF-1α has been proposed to act as a circulating mediator of RIc.^15^ However, treatment with a selective inhibitor of SDF-1α only partially attenuated but did not block RIc cardioprotection,^15^ suggesting the existence of other (parallel) mechanisms. Proteome/sequencing analysis also failed to reveal the nature of this factor.^34^^,^^45^^,^^46^ One recent study reported RIc-induced changes in plasma levels of seven proteins.^46^ These factors are involved in the control of haemostasis, lipid transport, iron regulation and inflammation. Some of the identified proteins (when applied exogenously) could mimic RIc in experimental models; however, it remains unknown whether blockade of their actions has an effect on RIc cardioprotection.

GLP-1 can activate GLP-1R expressed by visceral vagal afferents acting in a paracrine manner, but also has endocrine functions.^24^^,^^25^ Originally identified to be expressed by pancreatic β-cells, GLP-1R is now known to be widely distributed in many tissues, including the atrial myocardium, coronary vessels, and possibly, ventricles.^47^ Several studies demonstrated high efficacy of GLP-1R agonists in reducing the infarct size.^30^ However, the mechanisms by which GLP-1R activation protects the ischaemic myocardium remain largely unknown. Both GLP-1R-dependent and GLP-1R-independent mechanisms have been proposed.^28^^,^^29^ As the majority of the intestinally derived GLP-1 is degraded by the DPP-4, low levels of circulating GLP-1 are usually reported.^24^ Due to high rate of enzymatic degradation and renal clearance, GLP-1 has a short half-life (1–2 min) in plasma which represents a major limitation for its detection.^24^^,^^48^ In this study, we observed moderate increases in plasma GLP-1 level 30 min after the onset of the RIc stimulus, probably due to high variability of GLP-1 plasma concentration and short half-life of GLP-1. Interestingly, the long-lasting beneficial effects of short-term GLP-1 infusion have been shown to persist for weeks even when the circulating levels of GLP-1 return back to normal levels.^49^

The central finding of the present study is that highly selective GLP-1R antagonist Ex(9–39) administered before RIPre or RIPer blocks cardioprotection induced by both RIc stimuli. GLP-1R blockade also prevented the stimulatory effect of RIPre on AKT phosphorylation. It appears that different animal species recruit distinct cardioprotective signalling pathways. In rodents, RISK pathway (AKT) activation is essential to establish cardioprotection,^50^ but SAFE pathway (STAT3) also plays a role.^51^ In mice and rats, robust activation of RISK and SAFE pathways is observed in isolated hearts and *in vivo* preparations following application of various conditioning stimuli (RIPre and remote postconditioning) as well as pharmacologically.^11^^,^^50^^,^^52–56^ Importantly, there is also evidence that both RIc cardioprotection^52^ and cardioprotection induced by activation of GLP-1R^57^ are abolished in conditions of AKT blockade. In rodents, inhibition of either RISK or SAFE pathway blocks cardioprotection^11^^,^^52^^,^^54^^,^^56^ suggesting that the elements of these two protective pathways may interact. In pigs and humans, cardioprotection appears to be predominantly associated with activation of SAFE pathway.^58^^,^^59^ The data obtained in this study suggest that (at least in rats) GLP-1R-mediated signalling is essential for RIc cardioprotection by being responsible for triggering activation of the pro-survival RISK pathway and, therefore, GLP-1 is likely to act as the key humoral factor of this phenomenon.

### 4.4 The significance of cardiac vagal mechanisms in cardioprotection induced by GLP-1R activation

Potent cardioprotective effects of GLP-1R agonists have been demonstrated previously.^30^ However, the underlying mechanisms of GLP-1R-mediated cardioprotection remained largely unknown. Data showing that only atrial cardiomyocytes express GLP-1R,^47^ together with the evidence that GLP-1R agonists can establish cardioprotection in conditions of selective genetic GLP-1R deletion in ventricular cardiomyocytes,^29^ highlight major gaps in our understanding of GLP-1-induced cardioprotection. Here we addressed the potential mechanisms using systemic administration of a stable GLP-1R agonist Ex4, which is not readily cleaved by DPP-4. Cardioprotection established by Ex4 was found to be abolished in conditions of systemic muscarinic receptor blockade with atropine and, more specifically, M3 receptor blockade with 4-DAMP, whereas vagotomy had no effect. These data suggest that (most likely) cardiac M3-receptor mediated mechanisms are crucial for cardioprotection induced by Ex4. The hypothesised functions of cardiac M3 receptors include regulation of heart rate and cardiac repolarization, modulation of inotropic effects, regulation of cell-to-cell communication, and protection from ischaemia/reperfusion injury.^60^ We suggest that GLP-1 activates GLP-1R expressed by vagal efferent fibres innervating the ventricles, triggering pre-junctional release of acetylcholine, which protects ventricular cardiomyocytes via activation of M3 muscarinic receptors. Although, testing this hypothesis is beyond the scope of the present study, it is strongly supported by the recent data obtained by Pickard and colleagues showing that in isolated rat hearts, cardioprotection established by plasma dialysate from donor rats receiving RIc stimulus is abolished by muscarinic receptor blockade.^41^

### 4.5 Translational perspective

Results of several clinical studies demonstrated the efficacy of RIc in reducing infarct size in patients with AMI^4^^,^^5^ as well as improvement of long term prognosis in these patients.^6^ Other clinical reports demonstrated lack of RIc effect in patients undergoing cardiac surgery.^61^ In contrast, clinical studies which tested the efficacy of GLP-1R agonists in establishing cardioprotection demonstrated significant reductions in infarct size, regardless of comorbidities and prescribed medications.^31^^,^^32^ Results of the present study demonstrate that RIc cardioprotection is dependent on the actions of GLP-1, while cardioprotection induced by GLP-1R activation is independent of parasympathetic mechanisms but is mediated via M3 muscarinic receptor activation. These results provide a strong rationale for combination of RIc and intravenous GLP-1R agonists in patients with AMI.

## Supplementary material

Supplementary material is available at *Cardiovascular Research* online.

**Conflict of interest**: none declared.

## Funding

This work was supported by the British Heart Foundation (Ref: RG/14/4/30736), Medical Research Council (MR/N02589X/1) and The Wellcome Trust (Ref: 200893/Z/16/Z). A.V.G. is a Wellcome Trust Senior Research Fellow. S.M. is a Marie Skłodowska-Curie Research Fellow (Ref: 654691).

## Supplementary Material

Supplementary Data
