# Supplementary material for: Glucagon-like peptide-1 (GLP-1) mediates cardioprotection by remote ischaemic conditioning
Source: Cardiovasc Res. 2016 Oct 4;112(3):669–76. doi: 10.1093/cvr/cvw216 (PMC5157137; doi:10.1093/cvr/cvw216)
Supplement: Supplementary Data [file supp_cvw216_cvw216.DC1.html]

Supplementary Data | Cardiovascular Research

## Supplementary Data

files

- Supplementary Data - pdf file
