# Supplementary material for: Glucagon-like peptide-1 (GLP-1) mediates cardioprotection by remote ischaemic conditioning
Source: Cardiovasc Res. 2016 Oct 4;112(3):669–76. doi: 10.1093/cvr/cvw216 (PMC5157137; doi:10.1093/cvr/cvw216)
Supplement: Supplementary Data [file Supplementary_Table.pdf]

Supplementary Table

	VARIABLE	Baseline	Pre-ischaemia	End of ischaemia	Reperfusion (min)		
					30	60	120
Experiment 1							
Control (sham)	MAP	102±13	103±12	95±7	86±16	96±13	82±7
	HR	423±33	416±35	446±26	443±29	443±36	443±32
Cerv.vagotomy	MAP	99±15	98±13	94±19	99±21	101±22	93±18
	HR	425±29	436±34	463±36	461±31	455±30	455±25
Subd.vagotomy	MAP	101±14	105±12	101±16	102±17	104±14	100±19
	HR	418±38	423±29	431±30	435±30	434±29	437±23
RIPer	MAP	104±15	104±14	91±16	88±10	93±17	81±13
	HR	420±29	417±27	420±39	427±34	442±39	443±42
Cerv.vagotomy +RIPer	MAP	98±12	96±14	93±12	85±14	83±13	79±12
	HR	417±31	435±37	462±35	470±35	458±37	458±34
Subd.vagotomy +RIPer	MAP	99±14	98±12	99±7	89±8	87±9	77±9
	HR	406±29	463±21*	480±32*	487±38*	481±27*	492±28*
Experiment 2							
Control (sham)	MAP	101±11	96±12	90±6	85±8	89±7	85±12
	HR	417±37	421±33	427±37	433±31	436±27	435±35
IPre	MAP	98±11	99±18	98±12	91±10	85±9	86±13
	HR	432±26	444±16	438±19	435±22	441±19	444±22
Ex(9-39)+IPre	MAP	99±12	92±7	90±7	92±8	83±12	84±9
	HR	421±28	427±17	435±12	432±12	427±15	420±14
RIPre	MAP	100±12	92±10	98±11	94±11	97±9	88±13
	HR	428±37	430±34	438±30	412±30	412±34	410±32
Ex(9-39)+RIPre	MAP	97±12	98±14	81±14	81±13	78±14	84±7
	HR	415±22	425±31	434±33	424±35	431±30	445±18
RIPer	MAP	102±15	96±16	91±9	78±10	83±17	81±13
	HR	425±29	420±20	420±29	427±34	442±29	443±22
Ex(9-39)+RIPer	MAP	96±14	77±15	79±13	76±10	74±10	72±12
	HR	422±32	407±27	435±31	436±36	435±31	445±28
Experiment 3							
Control (sham)	MAP	97±11	97±13	81±19	80±10	83±13	89±15
	HR	409±27	427±28	418±27	418±24	415±31	445±38
Ex4	MAP	98±18	97±17	88±12	101±14	101±15	93±15
	HR	445±26	446±27	463±25	462±29	462±32	478±24*
Vagotomy+Ex4	MAP	97±9	125±12*	95±22	98±13	98±17	87±17
	HR	428±25	455±35*	475±31*	486±26*	502±34*	503±38*
Atropine+Ex4	MAP	101±7	93±9	88±8	81±7	79±9	80±9
	HR	432±15	431±36	447±29	447±38	453±33	450±38
4DAMP+Ex4	MAP	106±13	111±11	101±13	107±10	109±11	108±15
	HR	420±28	453±23	468±32	461±31	466±44	460±36

4DAMP – (1,1-Dimethyl-4-diphenylacetoxypiperidinium iodide); Ex(9-39) – Exendin (9-39); Ex4 – Exendin-4; HR – heart rate; IPre – ischaemic preconditioning; MAP – mean arterial blood pressure; RIPer – remote ischaemic perconditioning; RIPre – remote ischaemic preconditioning.
